# Drug Susceptibility, Siderophore Production, and Genome Analysis of *Staphylococcus aureus* Clinical Isolates from a University Hospital in Chiang Mai, Thailand

**DOI:** 10.3390/antibiotics14050521

**Published:** 2025-05-18

**Authors:** Warinda Prommachote, Manu Deeudom, Pimpisid Koonyosying, Phronpawee Srichomphoo, Ratchanee Somnabut, Phadungkiat Khamnoi, Agostino Cilibrizzi, Yuvaraj Ravikumar, Somdet Srichairatanakool

**Affiliations:** 1 Department of Biochemistry, Faculty of Medicine, Chiang Mai University, Chiang Mai 50200, Thailand; warinda.pr@wu.ac.th (W.P.); pimpisid.k@cmu.ac.th (P.K.); phronpawee0402@gmail.com (P.S.); 2 Faculty of Associated Medical Sciences, Walailak University, Nakhonsrithammarat 80160, Thailand; 3 Division of Bacteriology, Department of Microbiology, Faculty of Medicine, Chiang Mai University, Chiang Mai 50200, Thailand; manu.deeudom@cmu.ac.th; 4 Clinical Microbiology Laboratory, Maharaj Nakorn Chiang Mai Hospital, Faculty of Medicine, Chiang Mai University, Chiang Mai 50200, Thailand; ratchanee.so@cmu.ac.th (R.S.); micromedcmu@hotmail.com (P.K.); 5 Institute of Pharmaceutical Sciences, King’s College London, London SE1 9NH, UK; agostino.cilibrizzi@kcl.ac.uk; 6 Department of Biotechnology, Acharya Institute of Technology, Soladevanahalli, Karnataka 560170, India; yuvaraj3020@acharya.ac.in

**Keywords:** siderophore, iron, *Staphylococcus*, staphyloferrin, genome, DNA sequencing

## Abstract

**Background/Objective:***Staphylococcus aureus* produces staphyloferrin A (Sfna) siderophores to sequester host iron during infection and rapid cell proliferation We examined drug susceptibility, siderophore production, and genome sequencing of clinical isolates of *S. aureus*. **Methods:** A total of 100 specimens, including pus, sputum, hemoculture, urine, tissue, fluid, and skin scrap specimens, were grown in iron-deprived Luria broth agar. The isolates were investigated for spectral signature using MALDI–TOF/MS, while antibiotic susceptibility and siderophore content were assessed using the chrome azurol S method. Whole genome and partial 16S rRNA DNA sequences were employed, and VITEK/MS revealed specific spectra. **Results:** Clindamycin, erythromycin, gentamicin, linezolid, moxifloxacin, oxacillin, trimethoprim/sulfamethoxazole, and vancomycin (100%) were the most common antibiotics to which the *S. aureus* isolates were susceptible. Sfna was not detectable in fluid and skin scrap isolates, which were encoded by *sfnaB*, *sfnaD*, and *sfnaB*/*sfnaD* genes. However, they were detectable in pus (73.8%), sputum (85.3%), hemoculture (50.0%), and urine (85.7%) isolates. The aureus subspecies, JKD6159, SA268, and MN8, were found to be 72.73% according to genome sequencing. **Conclusion:** most staphylococci in the isolates, including *S. aureus* JKD6159, SA268, and MN8, were sensitive to antibiotics and were detected by MALDI–TOF/MS, resulting in the production of Sfna encoded by *sfna* genes.

## 1. Introduction

*Staphylococcus aureus* is an opportunistic Gram-positive cocci commonly present in human skin and mucous membranes of the respiratory tract, causing enterotoxin-induced food poisoning, foodborne gastroenteritis, skin ulcers, lethal sepsis, pneumonia, and septicemia [1,2,3,4]. *Staphylococcal* infections raise significant public health concerns as they have caused death among many patients. However, antibiotics can be used to treat these infections. Nevertheless, the infections can eventually become resistant to some drugs [5,6], and many of them can become less susceptible to drug treatments, causing methicillin-resistant *S. aureus* (MRSA), vancomycin-resistant *S. aureus* (VRSA) [7,8], and community-acquired methicillin-resistant *S. aureus* (CA–MRSA) [7,9,10]. Interestingly, *S. aureus* growth from clinical isolates was inhibited by a combination of bacterial siderophores, such as desferrioxamine (DFO), but could be abolished with ferric citrate [11]. In addition, ethylenediamine tetraacetic acid (EDTA) at an equivalent of 1 mM was more potent than DFO in significantly inhibiting *S. aureus* growth, while 2.5 μM DFO was found to enhance its growth [12].

Catalase (CAT) and coagulase tests have been used to identify the *S. aureus* species [13]. A matrix-assisted laser desorption ionization–time of flight mass spectrometry (MALDI–TOF/MS) can detect unique biomolecules, intact bacterial signatures [14], and antibiotic-resistant bacteria by accessing the spectral database library of peptide mass fingerprints of a specific microbial [15]. More importantly, the technique can be used to identify all *Staphylococcus* spp. bacteria and discriminate between them at the genus and species levels [16,17].

Under iron limitations, bacteria produce siderophores to sequester iron from hosts for their rapid proliferation and development [18]. Siderophores are low-molecular-weight molecules encoded by microbial-specific genomes that have a high binding affinity to complexes with ferric ions (Fe^3+^) with a high stability constant. Subsequently, the chelate exploits specific receptor channels on the cell membrane to traverse the cytoplasm, which is reduced to ferrous ions (Fe^2+^) through reductase enzymes, and enables bacteria to utilize soluble iron for survival [19]. Indeed, siderophores are required for maximal virulence, ensuring disruptions in the iron homeostasis of the host, increasing the bacterial distribution rate, causing drug resistance [20], and promoting colonization and tissue infection [21,22]. Siderophores, including hydroxamate, catecholate, carboxylate, and various mixed types, can be simply detected using the chrome azurol S (CAS) method [23]. In terms of their benefits, siderophores are used in the treatment of antibiotic-resistant bacteria, the inhibition of pathogen growth, and bacterial classification based on the secretory siderophore type [24].

For iron acquisition, *Staphylococcus* spp. produce and secrete two hydrophilic hexadentate carboxylate-type siderophores, known as staphyloferrin A (sfna), with a mass-to-charge (*m*/*z*) ratio of 479.12, and staphyloferrin B (sfnb), with an *m*/*z* value of 447.14 [25,26]. Due to carboxylate moieties, the siderophores favor a low pH environment (pKa values in a range of 3.5–5), and protonation allows the molecule to bind Fe^3+^ [27,28,29]. In competition, pathogenic *S. lugdunensis* combats the sfna and sfnb from *S. aureus* to acquire nonheme and heme iron from the host [30], for which the biosynthesis of sfna is encoded by the sfABCD operon and under the control of the Fe^3+^ uptake regulator (Fur) [31]. Within the bacterial heme transport system, the ATP synthase-binding cassette (ABC) transporter serves as a specific receptor for sfna, which comprises D–ornithine and two citrate molecules bound together with amide bonds on both sides. Sfna synthesis occurs through a non-ribosomal peptide synthetase-independent pathway [19,32]. Partial sequencing of the 16S ribosomal ribonucleic acid (rRNA) gene serves as a gold standard in the identification of the *Staphylococcus* species, but it is relatively time-consuming and requires high-quality DNA sequencing [17,33]. *Staphylococcus* bacteria have many cluster groups based on the 16S rRNA gene sequence [34]; thus, VITEK MALDI–TOF/MS has been recently used for the speciation of *S. aureus* and its discrimination in clinical isolates. Moreover, specific polymerase chain reaction (PCR) and genome sequencing assays have been used to analyze genetic diversity and virulence factors. Therefore, they have been employed to track the dissemination of *Staphylococcus* spp. infections [35,36]. It has been hypothesized that siderophores, particularly staphyloferrin, are secreted by *Staphylococcus* bacteria to chelate and utilize host iron during invasive infections, which can contribute to their antimicrobial activity and virulence. In the present study, we examined *Staphylococcus* isolates that were recovered from different patients, analyzed the secretory siderophores, evaluated drug susceptibility, and compared the VITEK MS-based species identification results with the data obtained from the 16S rRNA genome sequences.

## 2. Results

### 2.1. Specimen Collection and Biochemical Tests

A total of 100 *Staphylococcus* spp. specimens were isolated from patients (69 male and 31 female), including pus (n = 42), sputum (n = 33), hemoculture (n = 11), urine (n = 9), tissues (n = 3), fluid (n = 1), and skin scrap (n = 1), at the Clinical Microbiology Laboratory, Maharaj Nakorn Chiang Mai Hospital, Faculty of Medicine, Chiang Mai University, Chiang Mai, Thailand, between December 2020 and February 2022 (Table 1 and Supplementary Table S1). In addition, all the isolates showed positive results in CAT and coagulase tests (Table 1 and Supplementary Table S2).

### 2.2. MALDI–TOF MS Identification of S. aureus

**Figure 1:**
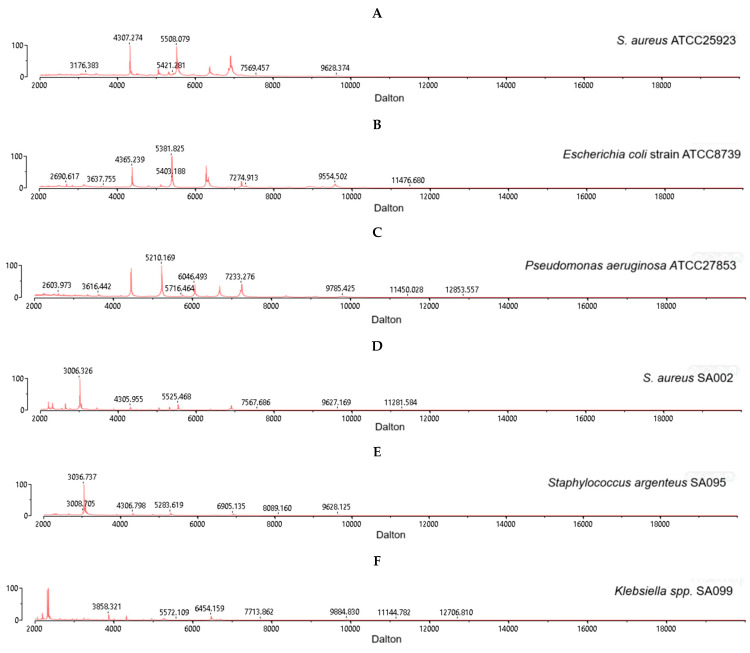
Spectra signatures obtained from VITEK 2 MALDI–TOF MS for *S. aureus* strain ATCC 25923 (**A**), *E. coli* strain ATCC 8739 (**B**), *P. aeruginosa* strain ATCC 27853 (**C**), *S. aureus* isolate SA002 (**D**), *S. argenteus* isolate SA095 (**E**), and *Klebsiella* spp. isolate SA099 (**F**).

All three reference strains of *S. aureus* ATCC 25923 (Figure 1A), including *Escherichia coli* strain ATCC25922 (Figure 1B) and *Pseudomonas aeruginosa* strain ATCC27853 (Figure 1C), were correctly identified by using the BioMerieux VITEK 2 MALDI–TOF MS system with a high confidence value (99.9%) and *m*/*z* values ranging from 2000 to 20,000 daltons. Among a total of 100 clinical isolates, *S. aureus* isolate SA002 (Figure 1D), *S. argenteus* isolate SA095 (Figure 1E), and *Klebsiella* spp. isolate SA099 (Figure 1F) were identified. In addition, our analysis has provided the spectral signatures of *S. aureus* (n = 94, SA001–SA037, SA039, SA041–SA056, SA058–SA063, SA065–SA094, SA096–SA098, and SA100), *S. argenteus* (n = 5, SA038, SA040, SA057, SA064, and SA095), and *Klebsiella* spp. (n = 1, SA099) (Supplementary Table S1 and Figure S1).

### 2.3. Drug Susceptibility Test for S. aureus Isolates

*S. aureus* can cause problems related to multidrug resistance (MDR) and virulence in many infections. Therefore, a panel of frontline antibiotics with different modes of action, including trimethoprim/sulfamethoxazole and vancomycin, is being routinely tested to inform antibiotic stewardship. In addition to methicillin, vancomycin has been reported to be resistant to *S. aureus* infections. Herein, *Staphylococcus* spp. isolates were subjected to susceptibility testing for these drugs. The MIC values for each antibiotic, including clindamycin, erythromycin, gentamycin, linezolid, moxifloxacin, oxacillin, trimethoprim/sulfamethoxazole, and vancomycin, were obtained from bacterial isolates. The results of the drug susceptibility assessments of one hundred *S. aureus* isolates are indicated in Table 2 and Supplementary Table S3, in which most of the isolates exhibited a high rate of susceptibility to the tested antibiotics (*p* < 0.05). For example, the isolates SA003, SA008, SA011, SA014, SA024, SA029, SA043, SA048, SA057, SA062, SA064, SA071, SA081, SA087, SA091, SA096, SA099, and SA100 were resistant to clindamycin and erythromycin. The isolate SA070 showed intermediate resistance to erythromycin. Likewise, the isolates SA002, SA003, and SA081 were resistant to gentamycin. The isolates SA002, SA004, SA011, SA048, SA071, and SA096 were resistant to moxifloxacin. The isolate SA028 was resistant to trimethoprim/sulfamethoxazole. Moreover, the isolates SA002, SA004, SA011, SA029, SA043, SA048, SA057, SA071, SA081, SA087, SA096, and SA099 were detected as MRSA bacteria. The remaining 22 isolates exhibited drug resistance patterns. However, none were resistant to linezolid or vancomycin.

### 2.4. Siderophore Production from S. aureus Isolates

**Figure 2:**
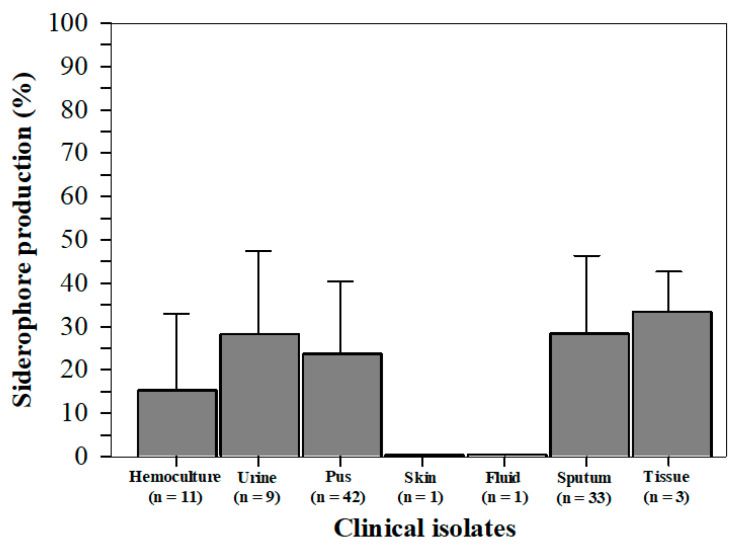
The percentage of *Staphylococcal* siderophore activity among hemoculture, urine, pus, skin, fluid, sputum, and tissue isolates (n = 100). Data are shown in individual values and mean ± SD values of two separate triplicate experiments.

We tested siderophore activity using a CAS assay. Siderophores were produced chiefly in tissue isolates (33.4 ± 9.3%); moderately (28.3 ± 19.1, 28.3 ± 18.0, and 23.7 ± 16.8%) in urine, sputum, and pus isolates; minutely in hemoculture (15.3 ± 17.8%), but not at all in skin and fluid isolates (Figure 2). The percentage of siderophore production of each isolate is also presented in Table S4.

### 2.5. Genomic DNA and Staphyloferrin A Biosynthesis Gene

**Figure 3:**
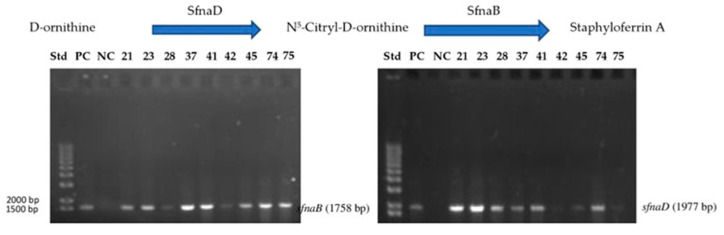
Agarose gel electrophoresis of PCR product for detection of *sfnaB* and *sfnaD* genes isolated from genomic DNA of *S. aureus* isolates 21, 23, 28, 37, 41, 42, 45, 74, and 75, including PC and NC samples. Abbreviations: DNA = deoxyribonucleic acid, NC = negative control, PC = positive control, PCR = polymerase chain reaction.

From polymerase chain reaction (PCR) analysis, the band for the *sfnaB* gene was detected in 34 samples, while the band for the *sfnaD* gene was detected in 18 samples. Here, the two genes were detected in 9 samples and labeled with the following numbers: 21, 23, 28, 37, 41, 42, 45, 74, and 75 (Figure 3).

### 2.6. Whole Genome Sequences

**Figure 4:**
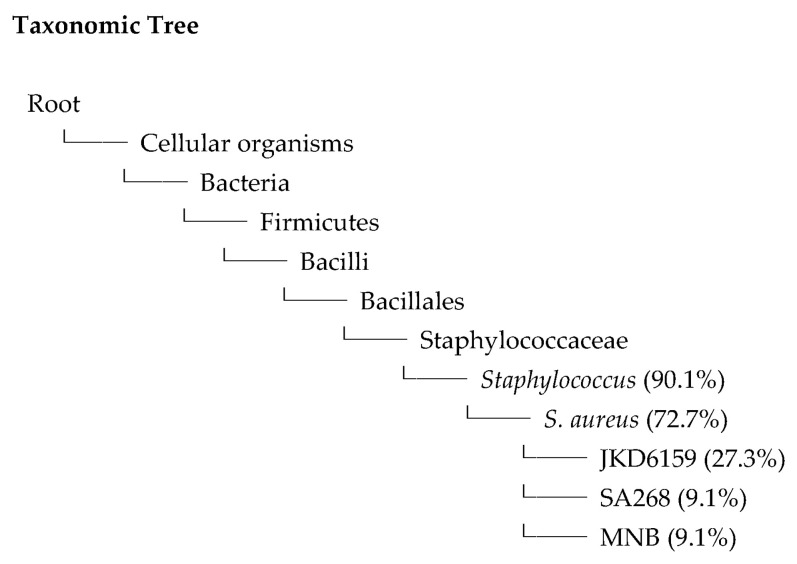
Staphylococcal taxonomic tree based on *sfna* and 16S rRNA genes. The nucleotide sequences of 16S rRNA obtained from *Staphylococci* were retrieved from GenBank. Note: relative frequency: the percentage of total sequences that match this taxon; absolute frequency: the number of reads or sequences assigned to that taxon; role: the code indicating the taxonomic rank (e.g., root, domain, genus, species, strain); taxon ID: the National Center for Biotechnology Information taxonomy identifier for that group; taxon, species, strain: the scientific name assigned to that taxon.

From the phylogenetic analysis shown in Table 3 and Figure 4, *S. aureus* isolates obtained from the patient’s sputum (isolate SA041) revealed a sequence homology close to *S. aureus* (72.7%): *S. aureus* subspeciesJKD6159 (27.3%) to *S. aureus* SA268 (9.1%) and *S. aureus* MN8 (9.1%).

**Figure 5:**
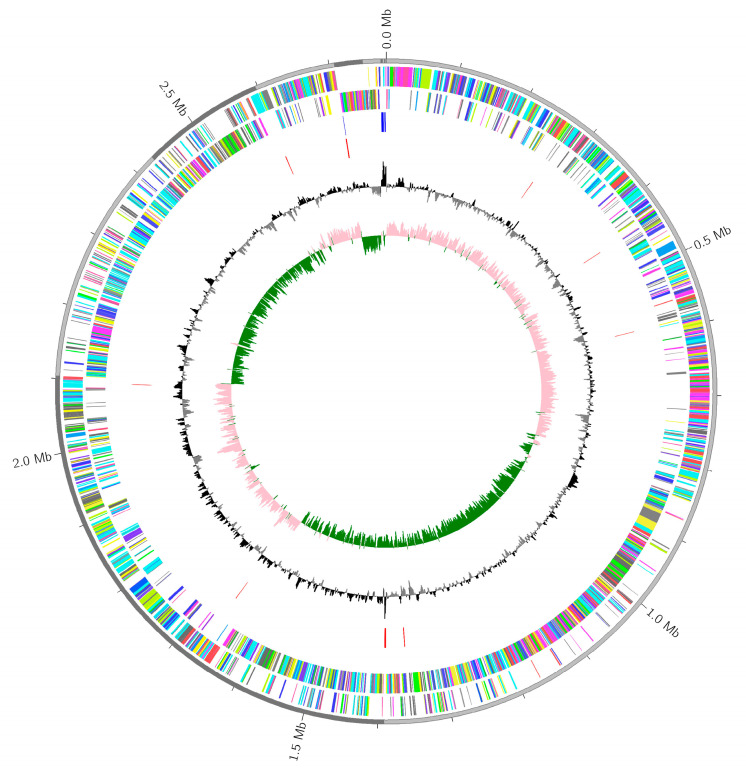

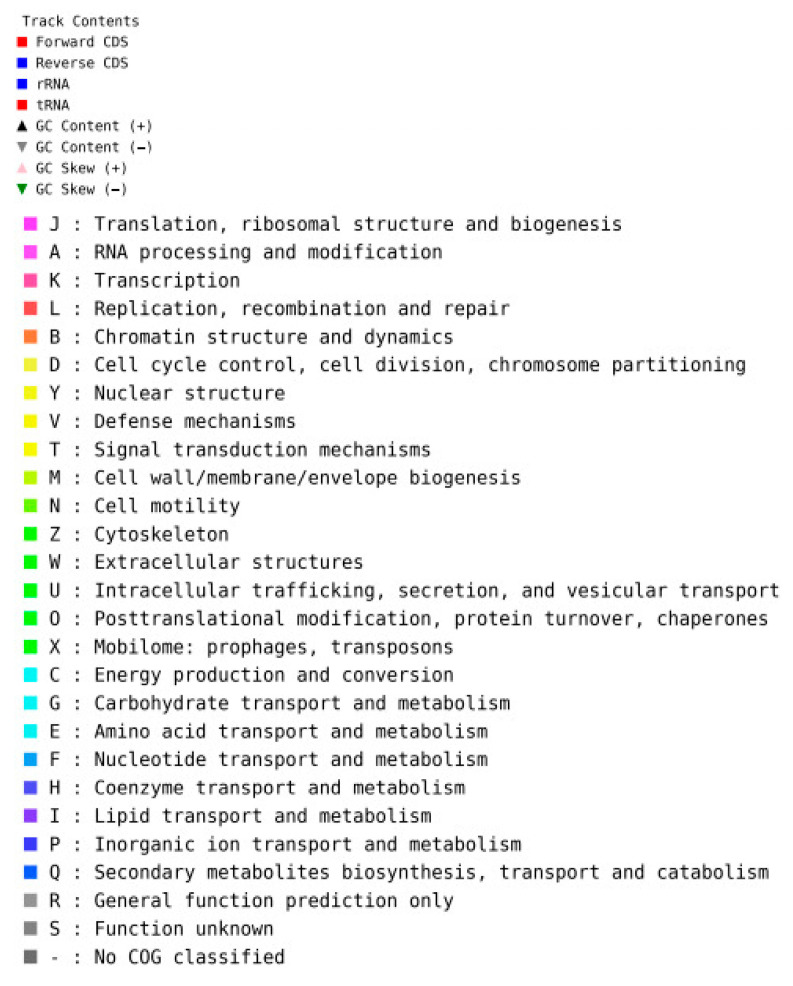
Plot results of the *Staphylococcus aureus* genome.

Each cluster of the orthologous groups (COG) of proteins is assembled as descendants from the same gene in the ancestral genome. A single genome map has been presented, indicating all assembled contigs achieved from the whole genome sequencing results of *S. aureus* (Figure 5). The top-edged circle represents all the contigs in the kb scale. The second and third circles show the coding sequencing (CDS) reference genes on the forward and reverse strands that have been represented in various colors according to COG categories. In addition, guanine (G), cytosine (C) content, and GC skews are shown in the last inner circle, respectively.

## 3. Discussion

*Staphylococcus* spp. are pathogenic Gram–positive cocci that can be treated by many classes of antibiotics; however, incidences of drug resistance have been reported [7,8]. Under iron limitations, bacteria have produced siderophores for iron acquisition from hosts [18]. These siderophores have also led to maximal virulence, thereby increasing the rate of bacterial distribution and promoting colonization and infection of the tissue [21,22]. In this study, we have included a total of one hundred specimens obtained from male and female patients, of which siderophores were produced mainly in the tissue, moderately in the urine, sputum, and pus, and minutely in the hemoculture but not in the skin and fluid isolates. In addition, drug sensitivity results have shown that most of the *S. aureus* colonies that were present in clinical isolates were sensitive to the antibiotics used, including clindamycin, erythromycin, gentamycin, linezolid, moxifloxacin, oxacillin, vancomycin, and a combination of trimethoprim and sulfamethoxazole. Nonetheless, Rocha et al. investigated bacterial isolates obtained from uterus and vagina specimens and reported a low level of production of biofilms and siderophores by coagulase-negative MRSA. This suggests the potential influence of certain host-related factors other than bacterial virulence [37].

The worldwide increase in antibiotic resistance has highlighted the need for new potential drugs and iron chelators that can function against bacterial pathogens. A susceptibility test for DFO siderophores was previously developed as a simple diagnostic tool for the identification of *S. epidermidis* and *S. hominis* obtained from hemocultures and other fluid specimens, indicating 96.4% efficiency, 97.3% sensitivity, and 91.8% specificity [38,39]. Additionally, two DFO and fosfomycin disc assays reported 99.5% sensitivity and 99.2% positive predictor values for the identification of *S. epidermidis* and *S. hominis* among coagulase-negative *Staphylococcus* spp. isolates [40]. Likewise, DFO-supplemented Columbia agar was adopted for the detection of MRSA with 94% sensitivity and 91% specificity [41]. An investigation of clinical peritoneal dialysis fluid and commensal skin samples demonstrated that *S. epidermidis* isolates produced the siderophore SA rather than SB, implying that SA production would be a virulence factor for *Staphylococcal* pathogenesis [42]. Davidov and colleagues reported that enterobactin and salmochelin siderophores effectively inhibited the growth of MRSA isolates; inversely, the low-dose siderophores enhanced bacterial growth [6] suggesting that suitable concentrations could stimulate or suppress *S. aureus* growth. Interestingly, Chakraborty and colleagues have indicated that macrocyclic polyketides obtained from marine heterotrophic Shewanella algae extract exerted Fe^3+^-chelating and anti-infective activities against vancomycin-resistant *Enterococcus faecalis*, MRSA, *P. aeruginosa*, and *K. pneumonia*, possibly by combating bacterial siderophores [43]. Consistently, bacteria sensitive to commercially available antibiotics expressed genes (1000–1400 base-pairs) that were determined to be involved in the biosynthesis and production of siderophores (MZ222387 and MZ222388) [44].

In routine microbiological assays, conventional bacteria identification requires a long culturing time of 12–16 h, the separation of the colonies for 24 h, and the identification of the species for 24–48 h by employing coagulase and CAT tests. Herein, we used the VITEK/MS method to confirm the *S. aureus* species in clinical isolates and compared the results with the two biochemical tests. In molecular analysis, MALDI–TOF/MS can detect proteins and lipids that are released from cells, as well as intact bacterial signatures, illustrating the different MS peaks formed by different peptide and protein *m*/*z* values, while indicating particular species and even genotypes within each species [14]. In addition, it can be used to characterize ribosomal protein biomarkers and mutations in the antibiotic resistance of *E. coli* [45,46]. Despite a fast, convenient, and actionable method, VITEK MALDI–TOF/MS requires an expensive instrument and access to the spectral database library of peptide mass fingerprints of each specific bacteria to provide valuable diagnostic information [15]. Remarkably, this technique can identify *Staphylococcus* (97.2%)*, Streptococci* (97.8%), *Enterococci* (100%) [16,47], *Cronobacter*, and *Vibrio* isolates [47], both rapidly and correctly.

Production and activity of SA and SB siderophores can be considered a virulence factor for the pathogenesis of coagulase-negative and coagulase-positive staphylococci in environments and clinical isolates. For rapid screening, colorimetric CAS, Arnow and Csaky liquids, and agar methods have been used to detect siderophore production from bacterial colonies and clinical isolates. As a consequence, the *S. aureus* strain ATCC 6538 and *S. epidermidis* obtained from human peritoneal dialysate exhibited higher iron utilization system (IUS) activity and grew better than the *S. aureus* ATCC 25923 strain, suggesting that the IUS activity plays an important role in bacterial growth and pathogenesis [48]. Thus, *S. aureus* per se adapts to the habitat by increasing the expression of the genes facilitating the acquisition of iron via the iron-binding molecule SB (*sfnb*) and the heme consumption protein (*isd*) pathways [49]. *P. aeruginosa* and *S. aureus* are the most prevalent respiratory pathogens causing thick mucus and chronic polymicrobial lung infections in cystic fibrosis patients. Accordingly, the supernatant derived from the *P. aeruginosa* culture reduced the sensitivity of *S. aureus* growth to a frontline antibiotic vancomycin and protected *S. aureus* from cell wall-active antibiotics and protein synthesis inhibitors, possibly by the effects of *Pseudomonas* pyoverdine and pyochelin siderophores [50]. Consistently, pyoverdine exhibited anti-microbial activity and effectively decreased the growth of pathogenic *Acinetobacter baumannii*, *K. pneumoniae*, and *S. aureus* in a concentration-dependent manner [51]. In addition, certain virulence factors, including the staphyloxanthin pigment obtained from *S. aureus* and the catecholate-type enterobactin siderophores derived from *E. coli*, are secreted during neutrophil phagocytosis, for which only staphyloxanthin prevented the bacteria from the toxicities of hypochlorous acid and related chloramines [52]. Moreover, a recent study has reported that enterobactin and salmochelin S4 siderophores inhibited the growth of *S. aureus,* including MRSA, in clinical isolates [6]. Furthermore, the *S. aureus* FhuD2 gene and protein involved in iron–hydroxamate chelate uptake were upregulated in infected tissues and were required for staphylococcal dissemination and abscess formation [53].

Genome analysis can provide insight into how *S. aureus* adapts to the environment and infection areas. Biosynthetic gene clusters are consecutive gene subsets that are present in various organisms and employed to produce specialized metabolites, which are mostly the non-ribosomal peptide synthase type and can exhibit antibacterial, anticancer, and iron-chelating activities. PCR-based identification is suitable for slow-growing and non-cultivable bacteria present in specific samples (e.g., cerebrospinal fluid and plasma) and requires nucleic acids obtained from isolated colonies [54,55]. Bacterial phylogenetic analysis is conducted to determine the evolutionary relationships among bacterial species and genera based on their 16S rRNA gene sequences. Based on 16S rRNA identification of staphylococcal genome sequences, *S. chromogenes* and *S. simulans* were the most frequent species, and *S. aureus* was the second most prevalent species associated with clinical mastitis; whereas, *S. chromogenes*, *S. simulans*, *S. Xylosus*, *S. haemolyticus*, *S. cohnii*, *S. epidermidis*, *S. capitis*, *S. sciuri*, *S. gallinarum*, *S. warneri*, *S. equorum*, *S. saprophyticus*, *S. succinus*, *S. arlettae*, and *S. agnetis* were the most common species associated with subclinical mastitis [35]. Consistently, 16S rRNA genes of *S. aureus*, *S. epidermidis*, *S. caprae*, and *S. capitis* have a close relationship with those observed in human clinical isolates and share a lot of adhesion genes [56,57].

Taken together, the findings suggest that the virulence factor from *S. aureus*, such as the sfna compound encoded by the *sfna* gene, is produced to sequester extracellular iron for cell metabolism and proliferation. In terms of its advantages, high-throughput MALDI–TOF MS contributes to a decrease in the amount of time involved with biochemical assays that are used for identifying bacterial species, which can also effectively save on the costs of the tests and lower the mortality rates of patients. Predominantly, the VITEK MS platform is a rapid (<5 min) and accurate (>90%) method of detection for identifying bacterial species by analysis of the molecular spectra of specific biomarkers (e.g., lipids and peptides) obtained from bacterial cell compositions. Nonetheless, the results are limited by intragenomic heterogeneity and certain difficulties associated with the analysis of all isolates, as well as difficulties in distinguishing between related *Staphylococcus* spp. This study investigated siderophore production in only one hundred staphylococcal isolates of the patients’ specimens, *sfna* gene expression in nine isolates, and the complete genome sequence in one sputum isolate.

## 4. Materials and Methods

### 4.1. Chemicals and Reagents

Trisodium 5–[(3–carboxy–5–methyl–4–oxocyclohexa–2,5–dien–1–ylidene)(2,6–dichloro–3–sulfonatophenyl)methyl]–3–methyl–2–oxidobenzoate or CAS (Product number 199532), ferric chloride hexahydrate (FeCl_3_·6H_2_O) (Product number 31232, ≥99% pure), hexadecyltrimethylammonium bromide (HDTMA) (Product number H5882, >98% pure), ethylenediamine–di(o–hydroxyphenylacetic acid (EDDHA) (Product number E4135, 98% pure), and piperazine–N, N′–bis(2–ethanesulfonic acid (PIPES) (Product number P6757) were purchased from Sigma-Aldrich Chemicals Company (Saint Louis, MO, USA). Luria–Bertani (LB) broth (Catalog number AAJ75882A1), Tris–acetate ethylenediamine tetraacetic acid (TAE) (Catalog number AAJ63931K7), Tris–minimal succinate (TMS) broth (Catalog number sc-264476), and agar plates (Catalog number 0701) were obtained from Thermo-Fischer Scientific Inc., Waltham, MA, USA. Antimicrobial drugs, including clindamycin, erythromycin, gentamicin, linezolid, moxifloxacin, oxacillin, trimethoprim/sulfamethoxazole, and vancomycin, were obtained from a pharmacy located at Maharaj Nakorn Chiang Mai Hospital, Faculty of Medicine, Chiang Mai University, Chiang Mai, Thailand.

### 4.2. Ethics

An expedited review for the accession of the patient and the clinical specimen information was submitted to the Human Ethics Committee of the Faculty of Medicine, Chiang Mai University, Thailand, and acceptance was generously granted by Emeritus Professor Panja Kulapongs, MD., Chairman (Research ID: 0404. Study Code: BIO-2566–0405, Date of Approval: 2 February 2024).

### 4.3. Institutional Review Board Statement

The protocol for biological material transfer and laboratory investigations was submitted to the Institutional Biosafety Committee (IBC) of the Faculty of Medicine, Chiang Mai University (Reference number: CMUIBC0265001, Date: 10 February 2022). It was then approved by the IBC and authorized by Associate Professor Dr. Sirikarn Lumpakarn, MD., PhD., Chairman (Reference number: CMUIBC02015/2565, Date: 11 August 2022).

### 4.4. Specimen Collection and Staphylococcus Culturing

*Staphylococcus aureus* strain ATCC 25923 was the pathogenic indicator strain stored at −80 °C in 40% glycerol. A total of one hundred isolates were recovered from human clinical specimens at Maharaj Nakorn Chiang Mai Hospital, Faculty of Medicine, Chiang Mai University, from December 2020 to February 2022. According to the isolation method described in the United States Food and Drug Administration Bacterial Analytical Manual 2001, all isolates and *S. aureus* ATCC 25923 reference strains were cultured on LB agar plates overnight and identified for *S. aureus* bacteria using Gram staining/light microscopy. The selected *S. aureus* colonies were inoculated into modified iron-deprived LB media, as recommended by Lindsay et al. [58], in which ferric chloride was added to the media to achieve concentrations of 0–2 µM. Afterward, the bacteria suspension was adjusted with the culture medium to reach an optical density of 0.5 McFarland units and incubated at 37 °C in a 5% CO_2_ incubator (Heraeus HeraCell150, Heraeus Group, Hanau, Germany) via gentle shaking at 150 rpm. Finally, the *S. aureus* growth was monitored every 24 h by measuring the optical density (OD) value at 600 nm against the culture medium with a double-beam spectrophotometer (Model UV-1900i Plus, Shimadzu Corporation, Kyoto, Japan) [59]. The *S. aureus* inoculum was identified using the Gram staining/microscopy technique, coagulase test, catalase test, and imaging mass spectrometry, which will be described below. In addition, the *S. aureus* stocks were maintained in LB broth containing 20% (*v*/*v*) glycerol and kept in a deep freezer at −70 °C.

### 4.5. Biochemical Identification of S. aureus

The coagulase test was performed on overnight bacterial cultures by mixing them with rabbit EDTA plasma. Then, the mixture was incubated at 37 °C for 4–6 h, and clot formation was observed after 4 h, indicating a coagulase-positive reaction by *S. aureus*. No clot formation meant a coagulase-negative reaction and that the isolate was from some other species of *Staphylococcus* [33]. In the CAT assay, the LB culture was directly flooded with a few drops of 3% (*v*/*v*) hydrogen peroxide, and bubbles were immediately observed.

### 4.6. MALDI-TOF/MS Analysis of Microbial–Specific Protein

An automated VITEK MS V3.2 machine (bioMérieux SA., Marcy–I’Etoile, Lyon, France) was run by the reagent (Reference number 411071) and operated via MALDI–TOF/MS technology. The process incorporated a comprehensive FDA 510(k) Vitek MS database version 2 for the in vitro diagnosis of bacteria, fungi, and mycobacteria. The machine providing the protein profile of the *S. aureus* strain, the phenotype-related biomarkers, and the drug-resistant strains was operated according to the prescribed protocol and manufacturer’s instructions [60]. Briefly, *S. aureus* colonies of the isolates were randomly selected and streaked onto the LB medium, and the *E. coli* ATCC 8739 calibration strain was streaked onto de Man, Rogosa, and Sharpe (MRS) agar at a pH of 6.8. They were then supplemented with 0.05% (*w*/*v*) L-cysteine HCl and then incubated at 37 °C for 24 h. Each colony was then purified by cross-streaking, while a portion of the colony was applied to a spot on a VITEK MS–DS target slide. The VITEK MS–CHCA matrix (a saturated solution of cyano–4–hydroxycinnamic acid in 50% acetonitrile and 2.5% trifluoroacetic acid) (1 µL) was immediately added to the spots and allowed to dry. Finally, the slide was subjected to multiple laser shots inside the VITEK MS instrument, which was operated according to the manufacturer’s instructions and protocol. Via computer operation, VITEK MS Acquisition Station software (Vitek MS version 1.0.0) was used to set up the parameters and record the data according to the manual. For setting up parameters, an *m*/*z* range was optimized between 2000 and 20,000, spectra were recorded in the positive linear mode, and 200–500 laser shots were accumulated from each spot. VITEK MS was calibrated with the *Escherichia coli* strain ATCC8739 for the positive control, and the matrix alone was employed for the negative control. In terms of data acquisition and interpretation, the generated mass spectra displayed three views, while an average of the peaks was processed by the machine computer. The sophisticated spectrum classifier algorithm autonomously recognized the organism by matching the acquired peaks with the reference spectrum of each asserted species to identify *S. aureus*. A percentage probability (confidential value) was then calculated, and this number represents the similarity of specific peaks between the generated spectrum and the database spectrum. At a confidence score of >99%, the analytical results were expressed as 2300–3000 for highly reliable species identification, 2000–2299 for highly reliable genus identification, 1700–1999 for probable genus identification, and 0–1699 for no reliable identification. When a single unique pattern was not identified, a list of possible bacteria was reported as “low discrimination”, or the species/strain could not be determined within the scope of the database and was reported as “no identification”.

### 4.7. Drug Susceptibility Testing

Drug susceptibility of all isolates was determined using the Sensititre™ ARIS 2X System (Thermo Fisher Scientific, Waltham, MA, USA) according to the manufacturer’s instructions and in accordance with the Clinical and Laboratory Standards Institute (CLSI) M100 Guidelines (2021) published in: *Performance Standards for Antimicrobial Susceptibility Testing*, 31st ed., Wayne Carley, Science and Education Publishing, Newark, DE, USA) [61]. Bacterial colonies obtained from the purity plates were prepared to 0.5 McFarland in cation-adjusted Mueller–Hinton (CAMH) broth using a Sensititre nephelometer. The suspension was dispensed into the Sensititre™ standard inoculum tray, while an automated inoculator (Sensititre™ Auto–Inoculator) was used to transfer the suspension into preloaded Sensititre™ susceptibility plates containing dried concentrations of antibiotics. The inoculated microdilution plates were sealed with adhesive covers, incubated at 35 ± 1 °C for 16–24 h under ambient air, and read using the ARIS 2X system, which automatically detected minimum inhibitory concentration (MIC) values by scanning the bacterial growth in each well. Accordingly, the MIC value, which is the lowest concentration that inhibited the visible growth of the bacteria, was obtained for the determination of clindamycin (S ≤ 0.5 µg/mL, I = 1–2 µg/mL, R ≥ 4 µg/mL), erythromycin (S ≤ 0.5 µg/mL, I = 1–4 µg/mL, R ≥ 8 µg/mL), gentamicin, (S ≤ 4 µg/mL, I = 8 µg/mL, R ≥ 16 µg/mL), linezolid (S ≤ 4 µg/mL, R ≥ 8 µg/mL), moxifloxacin (S ≤ 0.5 µg/mL, I = 1 µg/mL, R ≥ 2 µg/mL), oxacillin (S ≤ 2 µg/mL, R ≥ 4 µg/mL), trimethoprim/sulfamethoxazole (S ≤ 2/38 µg/mL, R ≥ 4/76 μg/mL), and vancomycin (S ≤ 2 µg/mL, I = 4–8 µg/mL, R ≥ 16 µg/mL).

### 4.8. Detection of Siderophore Production by Staphylococcus Isolates

#### 4.8.1. Preparation of Siderophore

*S. aureus* strains were grown with aeration in TMS broth containing 0.1 μM EDDHA for 40 h at 37 °C. Cells were removed by centrifugation, and supernatants were lyophilized. The dried supernatant was extracted with methanol (one-fifth of the original supernatant volume), passed through Whatman No. 1 filter paper to remove any insoluble materials, and then rotary evaporated. The siderophore sample was solubilized in DI water to 5% of the original supernatant volume and subjected to CAS assay.

#### 4.8.2. Colorimetric CAS Assay

Siderophores can be detected in biological fluids, liquid, and agar media using a colorimetric CAS assay [23,62], in which the chelator sequesters the iron from a blue-colored Fe^3+^–CAS complex (λ_max_ 630 nm) and decreases the color intensity. In a fresh preparation of the CAS reagent, 6 mL of 10 mM HDTMA solution, 1.5 mL of 1 mM FeCl_3_ solution previously dissolved in 10 mM HCl, 7.5 mL of 2 mM stock CAS solution, and 4.307 mg of PIPES were mixed thoroughly. The working CAS reagent was then adjusted to a final pH value of 5.6 and stored in the dark in a refrigerator at 4 °C. In the assay, the bacterial supernatant (100 µL each) was first added to each well of a 96-well microplate, and the CAS reagent (100 µL each) was subsequently added. Finally, the mixture was incubated at room temperature for 4 h, and the OD value was measured at 630 nm against the CAS reagent using an ELISA microplate reader (Model: MB580, Zhengzhou Medbes International Trading Limited Company, Henan, China). The percentage of siderophore production unit (SPU) was calculated by using the following Formula (1):

SPU = 100 × (OD_R_ − OD_S_)/OD_R_ (1)

OD_R_ = optical density of CAS reagent measured at 630 nm, OD_S_ = optical density of CAS treated with the sample measured at 630 nm.

### 4.9. Identification of sfnaB and sfnaD Genes

#### 4.9.1. Genomic DNA Extraction

Firstly, the *S. aureus* LB medium (1 mL for each isolate) was centrifuged to sediment the cells, resuspended in 0.5 mL of deionized water (DI), boiled in a water bath at 100 °C for 5 min, shaken vigorously to ensure the disruption of the cell wall, and centrifuged at 13,000× *g* for 5 min [63]. Then, genomic DNA obtained from the pellet was extracted and purified using DNeasy Blood & Tissue kits (QIAGEN, Valencia, CA, USA) following the manufacturer’s protocol and instructions. The concentration of the purified DNA samples was measured at 260 nm optical density using a NanoDrop 1000 spectrophotometer (NanoDrop Technology, Rockland, DE, USA) and stored at −20 °C until further use [33].

#### 4.9.2. Polymerase Chain Reaction Amplification

**Figure 6:**
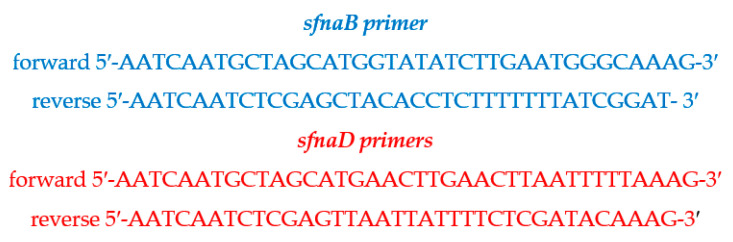
Primers used to identify *staphyloferrin A* subspecies *B* (*sfnaB*) and *D* (*sfnaD*) genes.

A fragment of the gene was amplified using sfnaB and sfnaD primer sets (Figure 6) for PCR and identification of the *sfnaB* and *sfnaD* genes [19].

Briefly, a total of 50 μL of the PCR reaction included 25 μL PCR HotStarTaq Master Mix (QIAGEN) and a 25 μL solution containing 200 nM of each primer, 1.5 mM additional MgCl_2_ (Promega, Madison, WI, USA), and 50 ng template DNA, which were diluted in PCR-grade water. QIAGEN HotStarTaq Master Mix solution containing HotStarTaq DNA polymerase, PCR buffer, and deoxyribonucleoside triphosphates (dNTPs) was diluted to reach a final concentration of 1.5 mM MgCl_2_ and 200 μM of each dNTP. The reactions were run for 30 cycles, of which each cycle was maintained at 95 °C for 3 min, 55.2 °C for 1 min, and 72 °C for 1 min in a GeneAmp PCR 9700 thermocycler (Applied Biosystems, Foster City, CA, USA) with an initial hot start (94 °C for 3 min) and a final extension step (72 °C for 6 min). The PCR-amplified product was electrophoresed on a 0.8% agarose gel at 100 volts for 30 min using TAE buffer and 1 kb plus DNA ladder markers (Invitrogen^TM^, Thermo-Fischer Scientific Inc., Waltham, MA, USA). It was then visualized under ultraviolet light exposure after staining [32].

#### 4.9.3. Nucleotide Sequencing and Data Analysis

The confirmed PCR-amplified products were sequenced using ABI BigDye v3.1 dye chemistry and AB 3500 XL automated DNA sequencers (Applied Biosystems, Foster City, CA, USA). To perform DNA sequencing, the PCR products were enzymatically cleaned before cycle sequencing, and 3 μL of ExoSAP–IT (USB Corporation, Cleveland, OH, USA) was added to the amplified PCR products (5 μL each). The mixture was then incubated at 37 °C for 20 min, followed by 80 °C for 15 min on the GeneAmp PCR 9700 thermocycler. The sequencing reactions contained cleaned PCR product (2 μL), BigDye Terminator v3.1 Ready Reaction Mix (1 μL), 5× sequencing buffer (2 μL), forward or reverse-sequencing primer (1.6 pmol), and water to a final volume of 20 μL. The sequencing reactions were subjected to 25 amplification cycles (each cycle was maintained at 96 °C for 30 s, 50 °C for 15 s, and 60 °C for 4 min) and held at 4 °C in a GeneAmp PCR 9700 thermocycler. Afterward, the reactions were cleaned up with Performa DTR Gel Filtration Cartridges (Edge Bio, Gaithersburg, MD, USA) according to the manufacturer’s instructions and protocol. The sequence accuracy of the DNA sequences was confirmed by two-directional sequencing. Multiple alignments of the nucleotide sequences were carried out using BioEdit, ClustalW, and Geneious programs version 7.2 (Informer Computer Systems, Inc., Garden Grove, CA, USA) with manual adjustments [32]. The generated nucleotide sequences of the partial 16S rRNA regions of all the *Staphylococcus* isolates were accessed and deposited in the GenBank database.

### 4.10. Whole Genome Sequencing and Phylogenetic Analysis

The putative staphyloferrin A-produced sample SA041 was employed to perform shotgun Illumina sequencing. The next-generation sequencing reads were first removed from primer sequences using Trim Galore version 0.6.7. Using a toolkit, quality control reads were then assembled de novo using a Saint Petersburg Genome Assembler (SPAdes) V3.13.0 and polished using Pilon version 1.23, which had been implemented in a Unicycler 0.5 [64]. Genome completeness was assessed using CheckM V1.2.1. Subsequently, Quast V5.2.0 was used to extract assembly statistics [65]. Genome visualization was performed using a microbial genome circular plotter. Gene calling and annotation were undertaken using Prokka V1.14.5. Accordingly, the predicted amino acid sequences were mapped to different functional categories, e.g., Kyoto Encyclopaedia of Genes and Genomes Orthology, Clusters of Orthologous Groups, and carbohydrate-active enzymes using eggNOG–mapper V–2.1.3 against the EggNog database 5.

### 4.11. Statistical Analysis

Data were collected and tabulated using Microsoft Excel software (version 16.75; Seattle, WA, USA). Descriptive analysis was performed using the Statistical Package for the Social Sciences Statistics version 21 for Windows (IBM Corporation, Armonk, NY, USA). The Shapiro–Wilk test was used to evaluate the distribution of normal or non-normal data. Quantitative variables were expressed as individual, percentage, and mean ± SD values. One-way analysis of variance with Tukey’s post hoc test for parametric data was used to compare the two groups to determine statistical significance, for which a *p*-value < 0.05 was considered statistically significant.

## 5. Conclusions

*Staphylococcus*-infected clinical isolates were positive for catalase and coagulase tests, of which MALDI–TOF MS signatures indicated 94% *S. aureus*. Most of the isolates were sensitive to the tested antibiotics. Sfna siderophores encoded by *sfnaD, sfnaB*, and *sfnaD/sfnaB* genes were produced chiefly in tissue, urine, sputum, pus, and hemoculture isolates, but not in the skin and fluid isolates. Genome sequence homology was mostly close to *S. aureus,* followed by subspecies JKD6159, SA268, and MN8. Further studies are needed involving more clinical samples to provide useful virulence profiling and a target for vaccine development against siderophore function during staphylococcal infections. The VITEK MALDI–TOF/MS instrument and the associated learning machines should be applied for rapid and accurate detection of staphyloferrins, enterobactin*,* salmochelin, pyoverdine, and aerobactin belonging to *S. aureus*, *E. coli*, *Salmonella typhimurium*, *P. aeruginosa*, and *K. pneumoniae*, respectively.

## Figures and Tables

**Table 1:** Types and biochemical tests of *Staphylococcus* spp. isolates obtained from patients (n = 100). Data are expressed in absolute and mean ± standard deviation (SD) values.

Specimen	Total (Female/Male)	Age (Years)	CAT	Coagulase
Pus	42 (19/23)	40.1 ± 21.1	+ (42)	+ (42)
Sputum	33 (8/25)	62.5 ± 21.8	+ (33)	+ (33)
Hemoculture	11 (2/9)	58.5 ± 17.7	+ (11)	+ (11)
Urine	9 (2/7)	63.2 ± 10.3	+ (9)	+ (9)
Tissue	3 (0/3)	47.3 ± 9.2	+ (3)	+ (3)
Fluid	1 (0/1)	33	+ (1)	+ (1)
Skin scrap	1 (0/1)	19	+ (1)	+ (1)

Abbreviation/symbol: CAT = catalase, + = positive.

**Table 2:** Antimicrobial susceptibility testing for the isolated colony obtained from different bacterial strains (n = 100). Briefly, bacterial colonies were suspended in cation-adjusted Mueller–Hinton broth, and the bacterial suspension was dispensed on Sensititre™ susceptibility plates containing dried concentrations of the antibiotics. The plates were then incubated at 37 °C overnight, and their drug susceptibility was determined.

Isolates	Antimicrobial Drugs							
	Clindamycin	Erythromycin	Gentamycin	Linezolid	Moxifloxacin	Oxacillin	Trimethoprim/Sulfamethoxazole	Vancomycin
Pus	35S, 7R	34S, 1I, 7R	42S	42S	38S, 4R	37S, 5R	41S, 1R	42S
Sputum	28S, 5R	28S, 5R	32S, 1R	33S	32S, 1R	29S, 4R	33S	33S
Hemoculture	8S,3R	8S,3R	10S,1R	11S	11S	10S,1R	11S	11S
Urine	7S, 2R	7S, 2R	8S, 1R	9S	8S, 1R	7S, 2R	9S	9S
Tissue	2S, 1R	2S, 1R	3S	3S	3S	3S	3S	3S
Fluid	1S	1S	1S	1S	1S	1S	1S	1S
Skin scarp	1S	1S	1S	1S	1S	1S	1S	1S

Abbreviations: I = intermediate, LB = Luria–Bertani (broth), R = resistant, S = sensitive.

**Table 3:** Relative and absolute frequency, as well as functions of *Staphylococcal* infections obtained from the complete genome analysis of *S. aureus* sputum isolate SA041.

Frequency				Identification
Relative	Absolute	Role	Taxon ID	Taxon, Species and Strain
100%	11	0 R	1	Root
100%	11	0 R1	131567	Cellular organism
100%	11	1 D	2	Bacteria
90.1%	10	0 D1	1783272	Terrabacteria group
90.1%	10	0 P	1239	Firmicutes
90.1%	10	0 C	91061	Bacilli
90.1%	10	0 O	1385	Bacillales
90.1%	10	0 F	90964	Staphylococcaceae
90.1%	10	2 G	1279	*Staphylococcus*
72.7%	8	3 S	1280	*Staphylococcus aureus*
27.3%	3	3 S1	869816	*Staphylococcus aureus* JKD6159
9.1%	1	1 S1	1368166	*Staphylococcus aureus* SA268
9.1%	1	1 S1	548470	*Staphylococcus aureus* MNB

Abbreviations: C = energy production and conversion, D = cell cycle control, F = nucleotide transport and metabolism, G = carbohydrate transport and metabolism, O = posttranslational modification, P = inorganic ion transport and metabolism, R = general function prediction, S = function unknown.

## Data Availability

All data presented in this study are available from the corresponding author upon reasonable request.

## Supplementary Materials

The following supporting information can be downloaded at: https://www.mdpi.com/article/10.3390/antibiotics14050521/s1, Table S1*:* Patients’ information, specimen types, microbiological and biochemical test results. Table S2: Identification of *Staphylococcus* spp. isolates (n = 100) by microbiological, biochemical, VITEK MS, and rRNA sequencing methods. Table S3: Drug susceptibility test for clinical isolates (n = 100). Table S4: Siderophore activity assayed in the clinical isolates (n = 100), including plus (n = 41), sputum (n =34), hemoculture (n = 11), urine (n = 9), tissue (n = 3), skin (n = 1), and fluid (n = 1). Figure S1: VITEK MS illustrations of *Staphylococcus* spp. isolates (SA001-SA100).

## Abbreviations

The following abbreviations have been used in this manuscript:

ABC	ATP synthase-binding cassette
C	Cytosine
CAMH	Cation-adjusted Mueller–Hinton (broth)
CAMRSA	Community-acquired methicillin-resistant *S. aureus*
CAS	Chrome azurol S
CAT	Catalase
CDS	Coding sequencing
CLSI	Clinical and Laboratory Standards Institute
COG	Cluster of orthologous groups
DFO	Desferrioxamine
DI	Deionized water
DNA	Deoxyribonucleic acid
dNTPs	Deoxyribonucleoside triphosphates
EDDHA	Ethylenediamine–di(o–hydroxyphenylacetic acid)
EDTA	Ethylenediamine tetraacetic acid
Fe^3+^	Ferric ion
Fe^2+^	Ferrous ion
G	Guanine
HDTMA	Hexadecyltrimethylammonium bromide
IBC	Institutional Biosafety Committee
IUS	Iron utilization system
LB	Luria–Bertani (broth)
MALDI–TOF/MS	Matrix-assisted laser desorption ionization–time of flight mass spectrometry
MIC	Minimal inhibitory concentration
MRS	Man, Rogosa and Sharpe
MRSA	Methicillin-resistant *S. aureus*
*m*/*z*	Mass-to-charge ratio
NC	Negative control
NIS	Non-ribosomal peptide synthetase
OD	Optical density
PC	Positive control
PCR	Polymerase chain reaction
PIPES	Piperazine–N, N′–bis(2–ethanesulfonic acid)
rRNA	Ribosomal ribonucleic acid
*S. aureus*	*Staphylococcus aureus*
SD	Standard deviation
sfna	Staphyloferrin A
sfnb	Staphyloferrin B
*S. epidermidis*	*Staphylococcus epidermidis*
*S. hominis*	*Staphylococcus hominis*
*S. hyicus*	*Staphylococcus hyicus*
*S. lugdunensis*	*Staphylococcus lugdunensis*
SPU	Siderophore production unit
TAE	Tris–acetate ethylenediamine tetraacetic acid
TMS	Tris–minimal succinate
T/S	Trimethoprim/sulfamethoxazole
VRSA	Vancomycin-resistant *S. aureus*
*v*/*v*	Volume by volume
*w*/*v*	Weight by volume
