# Supplementary material for: Drug Susceptibility, Siderophore Production, and Genome Analysis of Staphylococcus aureus Clinical Isolates from a University Hospital in Chiang Mai, Thailand
Source: Antibiotics (Basel). 2025 May 18;14(5):521. doi: 10.3390/antibiotics14050521 (PMC12108237; doi:10.3390/antibiotics14050521)
Supplement: Supplementary file 1 [file antibiotics-14-00521-s001.zip › antibiotics-3600923-supplementary.pdf]

Supplementary Information

Supplementary Tables

Table S1. Patients' information, specimen types, microbiological and biochemical test results.

Sample code	Age (year)	Gender	Type of specimens
SA001	66	M	Hemoculture
SA002	58	M	Urine from a catheter
SA003	76	M	Hemoculture
SA004	67	M	Pus from the sacrum wound
SA005	22	F	Pus axillary
SA006	53	M	Hemoculture
SA007	45	F	Pus from bartolin gland
SA008	82	M	Hemoculture
SA009	66	M	Pus
SA010	71	M	Hemoculture
SA011	70	M	Pus from the left hand
SA012	49	M	Pus from the left leg
SA013	55	F	Pus from right axilla
SA014	29	M	Pusfrom penis
SA015	19	F	Pus from the nail
SA016	19	M	Skin scrap lesion
SA017	33	M	Fluid from the spinal cord C4-C5
SA018	29	F	Pus from the right labia abscess
SA019	70	M	Urine
SA020	66	M	Hemoculture
SA021	87	F	Sputum
SA022	66	M	Sputum
SA023	25	M	Pus from a postoperative left femur wound
SA024	25	M	Pus from the abscess
SA025	68	M	Sputum
SA026	73	F	Sputum
SA027	88	M	Sputum
SA028	24	M	Pus
SA029	19	F	Pus from a postoperative head wound
SA030	70	M	Pus from the upper back
SA031	79	M	Pus from the left hand
SA032	61	M	Sputum
SA033	67	F	Urine
SA034	58	M	Tissue from the right thigh
SA035	21	M	Pus from the right armpit
SA036	62	M	Hemoculture
SA037	52	F	Pus from the left leg
SA038	22	F	Pus from the right forearm
SA039	80	M	Sputum
SA040	61	M	Sputum

SA041	42	M	Sputum
SA042	57	M	Sputum
SA043	29	F	Hemoculture
SA044	21	M	Sputum
SA045	40	M	Sputum
SA046	78	M	Sputum
SA047	79	F	Sputum
SA048	74	F	Sputum
SA049	87	M	Pus
SA050	44	M	Sputum
SA051	90	M	Sputum
SA052	22	M	Sputum
SA052	89	M	Sputum
SA054	75	M	Sputum
SA055	47	M	Urine
SA056	58	F	Urine
SA057	36	M	Sputum
SA058	86	F	Sputum
SA059	86	F	Sputum
SA060	22	F	Sputum
SA061	71	M	Sputum
SA062	28	M	Sputum
SA063	58	F	Pus spinal epidural
SA064	60	M	Urine
SA065	56	M	Urine
SA066	42	M	Pus from the left thigh
SA067	50	F	Pus
SA068	70	M	Pus from the left thigh
SA069	40	M	Pus from the right foot
SA070	25	M	Pus from the right thigh
SA071	34	M	Pus from the psoas abscess
SA072	26	M	Hemoculture 1
SA073	20	F	Pus abscess
SA074	44	M	Pus from the right maxillary sinus
SA075	71	M	Urine
SA076	78	M	Sputum
SA077	48	M	Sputum
SA078	61	M	Hemoculture
SA079	88	F	Pus
SA080	45	M	Sputum
SA081	72	M	Sputum
SA082	21	F	Pus from the right breast
SA083	20	F	Pus from the wound
SA084	44	F	Pus from the right breast

SA085	18	M	Pus from the right axillary
SA086	80	M	Sputum
SA087	41	F	Sputum
SA088	20	M	Pus from axillary
SA089	21	M	Pus from the abdominal wall
SA090	20	M	Pus from left-bit toe
SA091	42	M	Tissue+bone from left thigh
SA092	42	M	Tissue from the intramedullary canal
SA093	52	F	Hemoculture
SA094	24	F	Pus from the right ear
SA095	60	F	Pus from left arteriovenous fistula infection
SA096	19	F	Pus from the backhead
SA097	28	F	Pus skin wound
SA098	76	M	Sputum
SA099	82	M	Urine
SA100	42	M	Pus

Abbreviations: F = female, M = male.

Table S2. Identification of *Staphylococcus spp.* isolates(n = 100) by microbiological, biochemical, VITEK MS, and rRNA sequencing methods.

Sample code	Colony morphology	Biochemical test		VITEK MS		Confidence value (%)
		CAT	Coagulase	Genus	Species	
SA001	*Staphylococcus spp.*	+	+	*Staphylococcus*	*aureus*	>99
SA002	*Staphylococcus spp.*	+	+	*Staphylococcus*	*aureus*	>99
SA003	*Staphylococcus spp.*	+	+	*Staphylococcus*	*aureus*	>99
SA004	*Staphylococcus spp.*	+	+	*Staphylococcus*	*aureus*	>99
SA005	*Staphylococcus spp.*	+	+	*Staphylococcus*	*aureus*	>99
SA006	*Staphylococcus spp.*	+	+	*Staphylococcus*	*aureus*	>99
SA007	*Staphylococcus spp.*	+	+	*Staphylococcus*	*aureus*	>99
SA008	*Staphylococcus spp.*	+	+	*Staphylococcus*	*aureus*	>99
SA009	*Staphylococcus spp.*	+	+	*Staphylococcus*	*aureus*	>99
SA010	*Staphylococcus spp.*	+	+	*Staphylococcus*	*aureus*	>99
SA011	*Staphylococcus spp.*	+	+	*Staphylococcus*	*aureus*	>99
SA012	*Staphylococcus spp.*	+	+	*Staphylococcus*	*aureus*	>99
SA013	*Staphylococcus spp.*	+	+	*Staphylococcus*	*aureus*	>99
SA014	*Staphylococcus spp.*	+	+	*Staphylococcus*	*aureus*	>99
SA015	*Staphylococcus spp.*	+	+	*Staphylococcus*	*aureus*	>99
SA016	*Staphylococcus spp.*	+	+	*Staphylococcus*	*aureus*	>99
SA017	*Staphylococcus spp.*	+	+	*Staphylococcus*	*aureus*	>99
SA018	*Staphylococcus spp.*	+	+	*Staphylococcus*	*aureus*	>99
SA019	*Staphylococcus spp.*	+	+	*Staphylococcus*	*aureus*	>99
SA020	*Staphylococcus spp.*	+	+	*Staphylococcus*	*aureus*	>99
SA021	*Staphylococcus spp.*	+	+	*Staphylococcus*	*aureus*	>99
SA022	*Staphylococcus spp.*	+	+	*Staphylococcus*	*aureus*	>99

SA023	*Staphylococcus spp.*	+	+	*Staphylococcus*	*aureus*	>99
SA024	*Staphylococcus spp.*	+	+	*Staphylococcus*	*aureus*	>99
SA025	*Staphylococcus spp.*	+	+	*Staphylococcus*	*aureus*	>99
SA026	*Staphylococcus spp.*	+	+	*Staphylococcus*	*aureus*	>99
SA027	*Staphylococcus spp.*	+	+	*Staphylococcus*	*aureus*	>99
SA028	*Staphylococcus spp.*	+	+	*Staphylococcus*	*aureus*	>99
SA029	*Staphylococcus spp.*	+	+	*Staphylococcus*	*aureus*	>99
SA030	*Staphylococcus spp.*	+	+	*Staphylococcus*	*aureus*	>99
SA031	*Staphylococcus spp.*	+	+	*Staphylococcus*	*aureus*	>99
SA032	*Staphylococcus spp.*	+	+	*Staphylococcus*	*aureus*	>99
SA033	*Staphylococcus spp.*	+	+	*Staphylococcus*	*aureus*	>99
SA034	*Staphylococcus spp.*	+	+	*Staphylococcus*	*aureus*	>99
SA035	*Staphylococcus spp.*	+	+	*Staphylococcus*	*aureus*	>99
SA036	*Staphylococcus spp.*	+	+	*Staphylococcus*	*aureus*	>99
SA037	*Staphylococcus spp.*	+	+	*Staphylococcus*	*aureus*	>99
SA038	*Staphylococcus spp.*	+	+	*Staphylococcus*	*argenteus*	>99
SA039	*Staphylococcus spp.*	+	+	*Staphylococcus*	*aureus*	>99
SA040	*Staphylococcus spp.*	+	+	*Staphylococcus*	*argenteus*	>99
SA041	*Staphylococcus spp.*	+	+	*Staphylococcus*	*aureus*	>99
SA042	*Staphylococcus spp.*	+	+	*Staphylococcus*	*aureus*	>99
SA043	*Staphylococcus spp.*	+	+	*Staphylococcus*	*aureus*	>99
SA044	*Staphylococcus spp.*	+	+	*Staphylococcus*	*aureus*	>99
SA045	*Staphylococcus spp.*	+	+	*Staphylococcus*	*aureus*	>99
SA046	*Staphylococcus spp.*	+	+	*Staphylococcus*	*aureus*	>99
SA047	*Staphylococcus spp.*	+	+	*Staphylococcus*	*aureus*	>99
SA048	*Staphylococcus spp.*	+	+	*Staphylococcus*	*aureus*	>99
SA049	*Staphylococcus spp.*	+	+	*Staphylococcus*	*aureus*	>99
SA050	*Staphylococcus spp.*	+	+	*Staphylococcus*	*aureus*	>99
SA051	*Staphylococcus spp.*	+	+	*Staphylococcus*	*aureus*	>99
SA052	*Staphylococcus spp.*	+	+	*Staphylococcus*	*aureus*	>99
SA052	*Staphylococcus spp.*	+	+	*Staphylococcus*	*aureus*	>99
SA054	*Staphylococcus spp.*	+	+	*Staphylococcus*	*aureus*	>99
SA055	*Staphylococcus spp.*	+	+	*Staphylococcus*	*aureus*	>99
SA056	*Staphylococcus spp.*	+	+	*Staphylococcus*	*aureus*	>99
SA057	*Staphylococcus spp.*	+	+	*Staphylococcus*	*argenteus*	>99
SA058	*Staphylococcus spp.*	+	+	*Staphylococcus*	*aureus*	>99
SA059	*Staphylococcus spp.*	+	+	*Staphylococcus*	*aureus*	>99
SA060	*Staphylococcus spp.*	+	+	*Staphylococcus*	*aureus*	>99
SA061	*Staphylococcus spp.*	+	+	*Staphylococcus*	*aureus*	>99
SA062	*Staphylococcus spp.*	+	+	*Staphylococcus*	*aureus*	>99
SA063	*Staphylococcus spp.*	+	+	*Staphylococcus*	*aureus*	>99
SA064	*Staphylococcus spp.*	+	+	*Staphylococcus*	*argenteus*	>99
SA065	*Staphylococcus spp.*	+	+	*Staphylococcus*	*aureus*	>99
SA066	*Staphylococcus spp.*	+	+	*Staphylococcus*	*aureus*	>99

SA067	*Staphylococcus spp.*	+	+	*Staphylococcus*	*aureus*	>99
SA068	*Staphylococcus spp.*	+	+	*Staphylococcus*	*aureus*	>99
SA069	*Staphylococcus spp.*	+	+	*Staphylococcus*	*aureus*	>99
SA070	*Staphylococcus spp.*	+	+	*Staphylococcus*	*aureus*	>99
SA071	*Staphylococcus spp.*	+	+	*Staphylococcus*	*aureus*	>99
SA072	*Staphylococcus spp.*	+	+	*Staphylococcus*	*aureus*	>99
SA073	*Staphylococcus spp.*	+	+	*Staphylococcus*	*aureus*	>99
SA074	*Staphylococcus spp.*	+	+	*Staphylococcus*	*aureus*	>99
SA075	*Staphylococcus spp.*	+	+	*Staphylococcus*	*aureus*	>99
SA076	*Staphylococcus spp.*	+	+	*Staphylococcus*	*aureus*	>99
SA077	*Staphylococcus spp.*	+	+	*Staphylococcus*	*aureus*	>99
SA078	*Staphylococcus spp.*	+	+	*Staphylococcus*	*aureus*	>99
SA079	*Staphylococcus spp.*	+	+	*Staphylococcus*	*aureus*	>99
SA080	*Staphylococcus spp.*	+	+	*Staphylococcus*	*aureus*	>99
SA081	*Staphylococcus spp.*	+	+	*Staphylococcus*	*aureus*	>99
SA082	*Staphylococcus spp.*	+	+	*Staphylococcus*	*aureus*	>99
SA083	*Staphylococcus spp.*	+	+	*Staphylococcus*	*aureus*	>99
SA084	*Staphylococcus spp.*	+	+	*Staphylococcus*	*aureus*	>99
SA085	*Staphylococcus spp.*	+	+	*Staphylococcus*	*aureus*	>99
SA086	*Staphylococcus spp.*	+	+	*Staphylococcus*	*aureus*	>99
SA087	*Staphylococcus spp.*	+	+	*Staphylococcus*	*aureus*	>99
SA088	*Staphylococcus spp.*	+	+	*Staphylococcus*	*aureus*	>99
SA089	*Staphylococcus spp.*	+	+	*Staphylococcus*	*aureus*	>99
SA090	*Staphylococcus spp.*	+	+	*Staphylococcus*	*aureus*	>99
SA091	*Staphylococcus spp.*	+	+	*Staphylococcus*	*aureus*	>99
SA092	*Staphylococcus spp.*	+	+	*Staphylococcus*	*aureus*	>99
SA093	*Staphylococcus spp.*	+	+	*Staphylococcus*	*aureus*	>99
SA094	*Staphylococcus spp.*	+	+	*Staphylococcus*	*aureus*	>99
SA095	*Staphylococcus spp.*	+	+	*Staphylococcus*	*argenteus*	>99
SA096	*Staphylococcus spp.*	+	+	*Staphylococcus*	*aureus*	>99
SA097	*Staphylococcus spp.*	+	+	*Staphylococcus*	*aureus*	>99
SA098	*Staphylococcus spp.*	+	+	*Staphylococcus*	*aureus*	>99
SA099	*Staphylococcus spp.*	+	+	*Klebsiella*	-	>99
SA100	*Staphylococcus spp.*	+	+	*Staphylococcus*	*aureus*	>99

Abbreviations: CAT = catalase, MALDI–TOF/MS = matrix-assisted laser desorption ionization-time of flight mass spectrometry.

Table S3. Drug susceptibility test for clinical isolates (n = 100).

**Sample Code**	**Antimicrobial drugs**							
	Clindamycin	Erythromycin	Gentamycin	Linezolid	Moxifloxacin	Oxacillin	Trimethoprim/sulfamethoxazole	Vancomycin
SA001	S	S	S	S	S	S	S	S
SA002	S	S	R	S	R	R	S	S
SA003	R	R	R	S	S	S	S	S
SA004	S	S	S	S	R	R	S	S
SA005	S	S	S	S	S	S	S	S

SA006	S	S	S	S	S	S	S	S
SA007	S	S	S	S	S	S	S	S
SA008	R	R	S	S	S	S	S	S
SA009	S	S	S	S	S	S	S	S
SA010	S	S	S	S	S	S	S	S
SA011	R	R	S	S	R	R	S	S
SA012	S	S	S	S	S	S	S	S
SA013	S	S	S	S	S	S	S	S
SA014	R	R	S	S	S	S	S	S
SA015	S	S	S	S	S	S	S	S
SA016	S	S	S	S	S	S	S	S
SA017	S	S	S	S	S	S	S	S
SA018	S	S	S	S	S	S	S	S
SA019	S	S	S	S	S	S	S	S
SA020	S	S	S	S	S	S	S	S
SA021	S	S	S	S	S	S	S	S
SA022	S	S	S	S	S	S	S	S
SA023	S	S	S	S	S	S	S	S
SA024	R	R	S	S	S	S	S	S
SA025	S	S	S	S	S	S	S	S
SA026	S	S	S	S	S	S	S	S
SA027	S	S	S	S	S	S	S	S
SA028	S	S	S	S	S	S	R	S
SA029	R	R	S	S	S	R	S	S
SA030	S	S	S	S	S	S	S	S
SA031	S	S	S	S	S	S	S	S
SA032	S	S	S	S	S	S	S	S
SA033	S	S	S	S	S	S	S	S
SA034	S	S	S	S	S	S	S	S
SA035	S	S	S	S	S	S	S	S
SA036	S	S	S	S	S	S	S	S
SA037	S	S	S	S	S	S	S	S
SA038	S	S	S	S	S	S	S	S
SA039	S	S	S	S	S	S	S	S
SA040	S	S	S	S	S	S	S	S
SA041	S	S	S	S	S	S	S	S
SA042	S	S	S	S	S	S	S	S
SA043	R	R	S	S	S	R	S	S
SA044	S	S	S	S	S	S	S	S
SA045	S	S	S	S	S	S	S	S
SA046	S	S	S	S	S	S	S	S
SA047	S	S	S	S	S	S	S	S
SA048	R	R	S	S	R	R	S	S
SA049	S	S	S	S	S	S	S	S
SA050	S	S	S	S	S	S	S	S
SA051	S	S	S	S	S	S	S	S
SA052	S	S	S	S	S	S	S	S
SA052	S	S	S	S	S	S	S	S
SA054	S	S	S	S	S	S	S	S
SA055	S	S	S	S	S	S	S	S
SA056	S	S	S	S	S	S	S	S
SA057	R	R	S	S	S	R	S	S
SA058	S	S	S	S	S	S	S	S

SA059	S	S	S	S	S	S	S	S
SA060	S	S	S	S	S	S	S	S
SA061	S	S	S	S	S	S	S	S
SA062	R	R	S	S	S	S	S	S
SA063	S	S	S	S	S	S	S	S
SA064	R	R	S	S	S	S	S	S
SA065	S	S	S	S	S	S	S	S
SA066	S	S	S	S	S	S	S	S
SA067	S	S	S	S	S	S	S	S
SA068	S	S	S	S	S	S	S	S
SA069	S	S	S	S	S	S	S	S
SA070	S	I	S	S	S	S	S	S
SA071	R	R	S	S	R	R	S	S
SA072	S	S	S	S	S	S	S	S
SA073	S	S	S	S	S	S	S	S
SA074	S	S	S	S	S	S	S	S
SA075	S	S	S	S	S	S	S	S
SA076	S	S	S	S	S	S	S	S
SA077	S	S	S	S	S	S	S	S
SA078	S	S	S	S	S	S	S	S
SA079	S	S	S	S	S	S	S	S
SA080	S	S	S	S	S	S	S	S
SA081	R	R	R	S	S	R	S	S
SA082	S	S	S	S	S	S	S	S
SA083	S	S	S	S	S	S	S	S
SA084	S	S	S	S	S	S	S	S
SA085	S	S	S	S	S	S	S	S
SA086	S	S	S	S	S	S	S	S
SA087	R	R	S	S	S	R	S	S
SA088	S	S	S	S	S	S	S	S
SA089	S	S	S	S	S	S	S	S
SA090	S	S	S	S	S	S	S	S
SA091	R	R	S	S	S	S	S	S
SA092	S	S	S	S	S	S	S	S
SA093	S	S	S	S	S	S	S	S
SA094	S	S	S	S	S	S	S	S
SA095	S	S	S	S	S	S	S	S
SA096	R	R	S	S	R	R	S	S
SA097	S	S	S	S	S	S	S	S
SA098	S	S	S	S	S	S	S	S
SA099	R	R	S	S	S	R	S	S
SA100	R	R	S	S	S	S	S	S
**Total**	**82S/18R**	**81S/1I/18R**	**97S/3R**	**100S**	**94S/6R**	**88S/12R**	**99S/1R**	**100S**

Abbreviations: I = intermediate, R = resistant, S = sensitive.

Table S4. Siderophore activity assayed in the clinical isolates (n = 100) including plus (n = 41), sputum (n =34), hemoculture (n = 11), urine (n = 9), tissue (n = 3), skin (n = 1), and fluid (n = 1).

Sample Code	Siderophore activity (%)			
	**Batch 1**	**Batch 2**	**Mean±SD**	**Interpretation**
SA001	38.0	37.64	37.71±0.35	Positive
SA002	0.0	0.00	0	Negative

SA003	0.0	0.00	0	Negative
SA004	2.4	2.15	2.20±0.13	Positive
SA005	15.5	14.56	14.75±0.58	Positive
SA006	0.0	0.00	0	Negative
SA007	26.7	28.80	28.38±1.08	Positive
SA008	4.0	3.63	3.71±0.35	Positive
SA009	29.6	27.20	27.69±1.53	Positive
SA010	40.8	42.67	42.31±1.69	Positive
SA011	2.0	1.82	1.85±0.10	Positive
SA012	37.1	37.46	37.38±1.00	Positive
SA013	39.5	32.21	33.67±3.71	Positive
SA014	0.0	0.00	0	Negative
SA015	5.2	12.11	10.72±3.38	Positive
SA016	0.0	0.00	0	Negative
SA017	0.0	0.00	0	Negative
SA018	34.0	33.12	33.31±1.77	Positive
SA019	34.1	42.68	40.96±4.12	Positive
SA020	0.0	0.00	0	Negative
SA021	0.0	0.00	0.19±0.42	Negative
SA022	7.4	11.51	10.69±2.33	Positive
SA023	13.6	23.70	21.68±6.41	Positive
SA024	7.1	6.61	6.70±1.55	Positive
SA025	6.9	10.22	9.55±1.49	Positive
SA026	27.0	37.03	35.03±4.61	Positive
SA027	0.0	0.00	0	Negative
SA028	0.0	0.00	0	Negative
SA029	0.0	0.00	0	Negative
SA030	3.2	8.98	7.82±2.84	Positive
SA031	0.0	0.00	0	Negative
SA032	24.2	13.58	15.69±5.37	Positive
SA033	19.1	17.45	17.79±0.76	Positive
SA034	24.9	24.89	24.89±2.20	Positive
SA035	0.0	0.00	0.46±1.04	Negative
SA036	0.0	0.00	0	Negative
SA037	26.5	27.12	27.00±1.55	Positive
SA038	30.5	29.90	30.01±0.77	Positive
SA039	26.9	26.82	26.84±2.39	Positive
SA040	27.0	30.18	29.54±1.62	Positive
SA041	26.2	26.87	26.74±2.28	Positive
SA042	32.1	32.03	32.04±2.54	Positive
SA043	33.2	16.86	20.13±7.32	Positive
SA044	61.4	53.24	54.87±3.97	Positive
SA045	9.7	8.50	8.74±0.83	Positive
SA046	0.0	0.00	0	Negative
SA047	45.1	47.08	46.68±2.03	Positive
SA048	26.9	23.62	24.28±1.49	Positive
SA049	33.2	30.97	31.41±1.83	Positive
SA050	35.3	30.84	31.72±2.42	Positive
SA051	44.9	54.88	52.87±5.24	Positive
SA052	0.0	0.00	0	Negative
SA052	46.9	32.39	35.29±7.01	Positive
SA054	54.3	42.21	44.64 ±5.99	Positive
SA055	49.0	44.85	45.67±1.91	Positive

SA056	11.2	34.74	30.03±10.56	Positive
SA057	36.1	45.45	43.58±4.18	Positive
SA058	47.9	56.02	54.40±3.67	Positive
SA059	45.5	48.28	47.71±1.34	Positive
SA060	19.6	18.31	18.58±1.04	Positive
SA061	46.4	48.95	48.44±1.39	Positive
SA062	13.5	12.21	12.47±1.16	Positive
SA063	48.2	51.97	51.21±2.02	Positive
SA064	11.2	11.95	11.80±0.93	Positive
SA065	38.8	45.68	44.30±3.09	Positive
SA066	44.8	48.19	47.52±1.61	Positive
SA067	38.9	51.97	49.35±5.96	Positive
SA068	19.3	16.15	16.77±1.67	Positive
SA069	13.7	23.97	21.92±4.66	Positive
SA070	22.9	18.63	19.49±1.99	Positive
SA071	0.0	0.00	0	Negative
SA072	32.4	36.43	35.63±1.96	Positive
SA073	44.4	49.25	48.29±2.23	Positive
SA074	34.6	41.06	39.77±3.08	Positive
SA075	14.1	8.68	9.76±2.44	Positive
SA076	41.8	42.24	42.14±0.56	Positive
SA077	41.7	46.13	45.23±2.01	Positive
SA078	31.8	27.95	28.72±1.76	Positive
SA079	41.9	45.38	44.68±1.94	Positive
SA080	33.9	23.70	25.74±4.81	Positive
SA081	0.0	0.00	0	Negative
SA082	22.9	22.88	22.88±0.82	Positive
SA083	39.4	47.08	45.54±3.50	Positive
SA084	0.0	0.00	0	Negative
SA085	43.5	47.26	46.51±1.77	Positive
SA086	44.2	41.71	42.20±1.80	Positive
SA087	9.1	11.15	10.75±1.11	Positive
SA088	49.8	37.67	40.11±5.49	Positive
SA089	0.0	0.00	0	Negative
SA090	34.9	29.90	30.90±2.37	Positive
SA091	42.2	43.63	43.34±1.18	Positive
SA092	38.7	30.13	31.85±3.87	Positive
SA093	0.00	0.00	0	Negative
SA094	31.8	34.09	33.63±1.17	Positive
SA095	45.1	43.22	43.60±3.37	Positive
SA096	25.9	22.13	22.89±1.71	Positive
SA097	30.9	33.57	33.04±1.22	Positive
SA098	45.4	42.94	43.43±1.38	Positive
SA099	49.1	55.64	54.33±2.92	Positive
SA100	40.9	43.35	42.86±1.24	Positive

Supplementary Figure

Figure S1. VITEK MS illustrations of *Staphylococcus spp.* isolates (SA001-SA100).

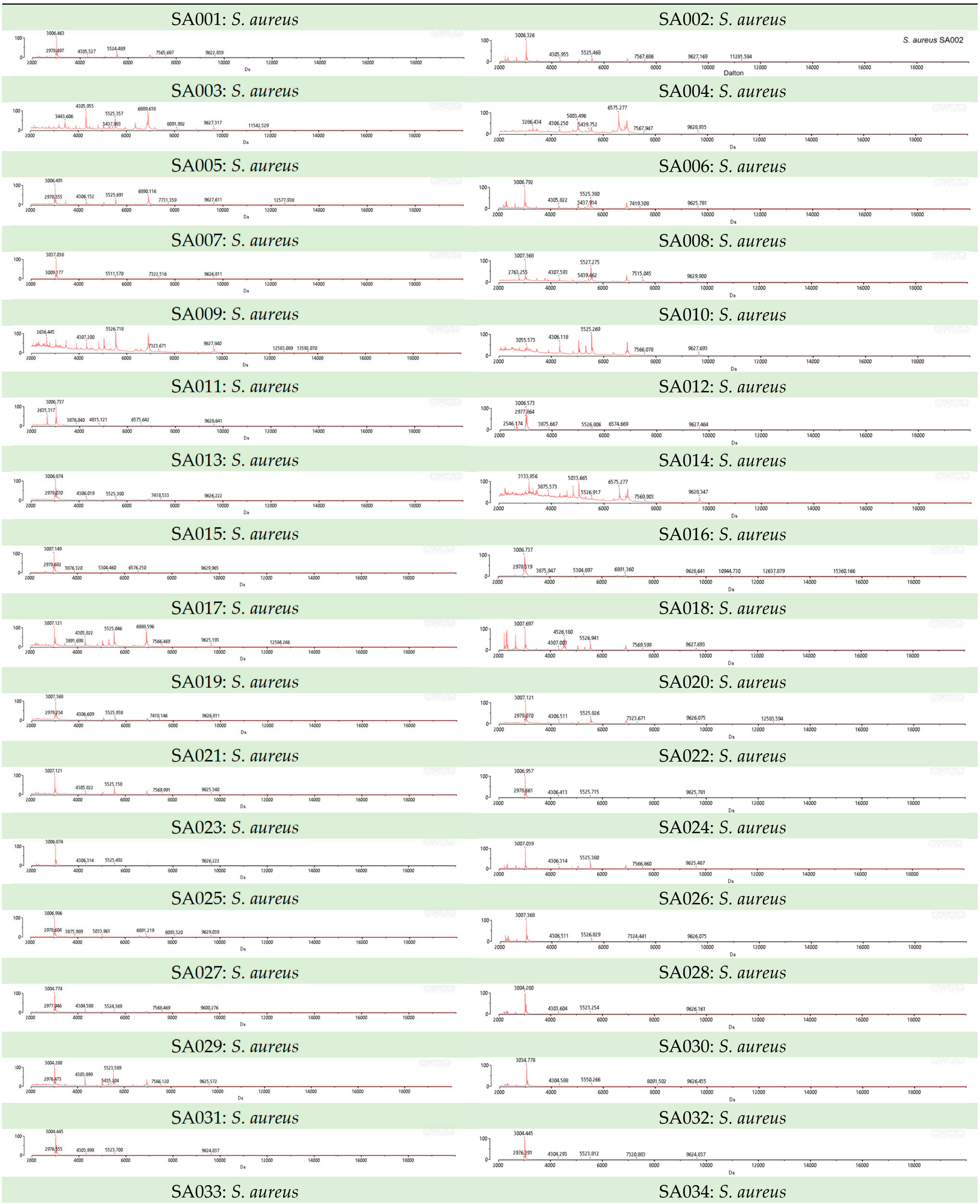

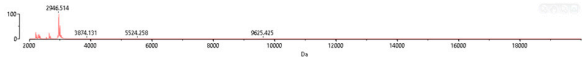

SA035: *S. aureus*

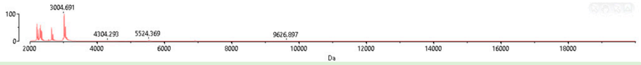

SA036: *S. aureus*

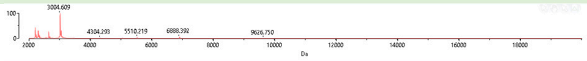

SA037: *S. aureus*

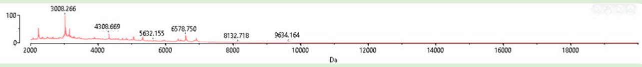

SA038: *S. argenteus*

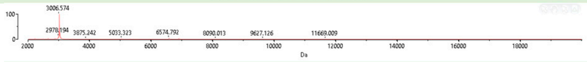

SA039: *S. aureus*

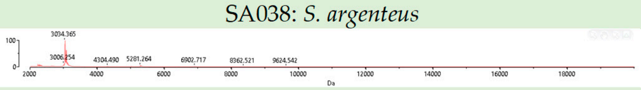

SA040: *S. argenteus*

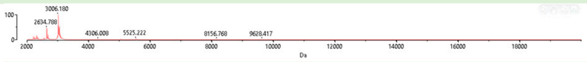

SA041: *S. aureus*

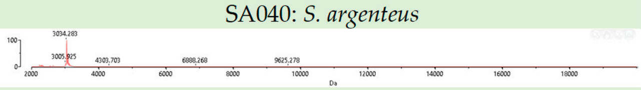

SA042: *S. aureus*

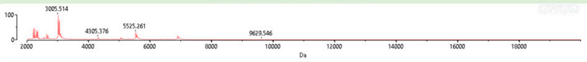

SA043: *S. aureus*

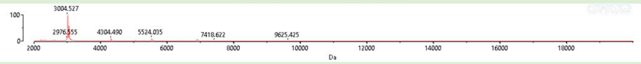

SA044: *S. aureus*

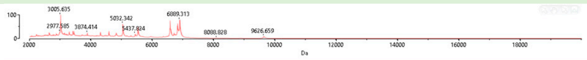

SA045: *S. aureus*

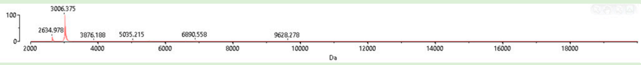

SA046: *S. aureus*

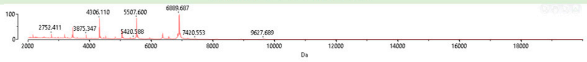

SA047: *S. aureus*

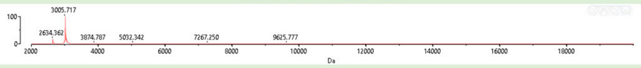

SA048: *S. aureus*

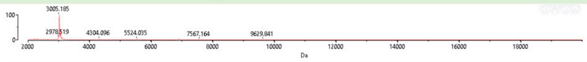

SA049: *S. aureus*

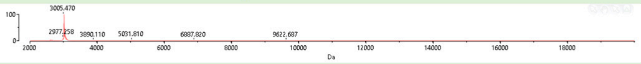

SA050: *S. aureus*

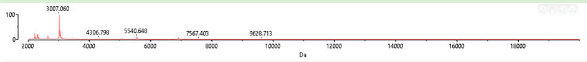

SA051: *S. aureus*

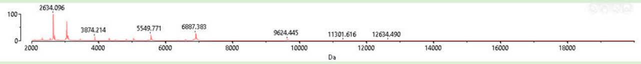

SA052: *S. aureus*

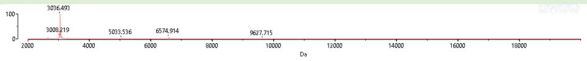

SA053: *S. aureus*

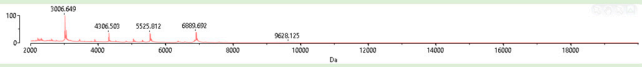

SA054: *S. aureus*

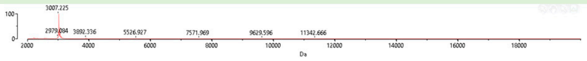

SA055: *S. aureus*

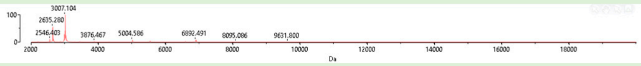

SA056: *S. aureus*

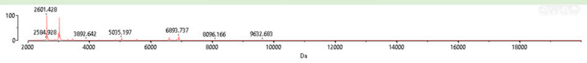

SA057: *S. argenteus*

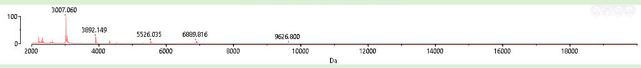

SA058: *S. aureus*

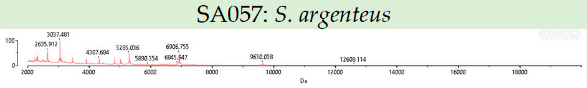

SA059: *S. aureus*

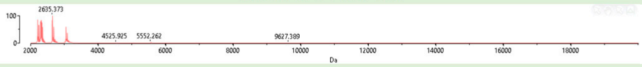

SA060: *S. aureus*

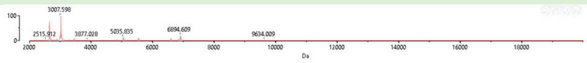

SA061: *S. aureus*

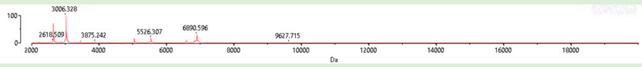

SA062: *S. aureus*

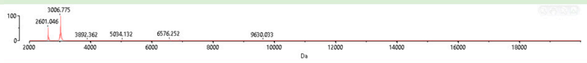

SA063: *S. aureus*

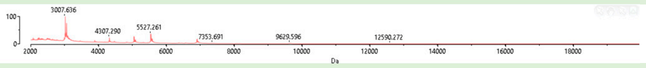

SA064: *S. argenteus*

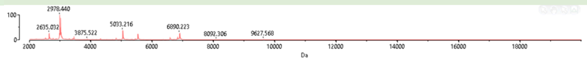

SA065: *S. aureus*

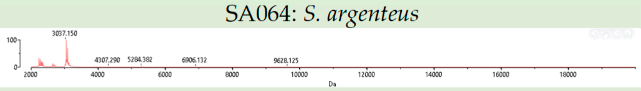

SA066: *S. aureus*

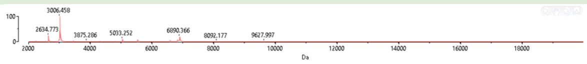

SA067: *S. aureus*

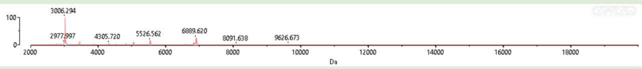

SA068: *S. aureus*

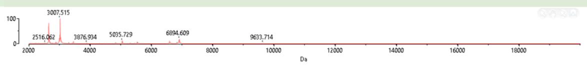

SA069: *S. aureus*

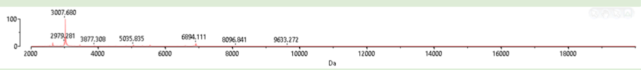

SA070: *S. aureus*

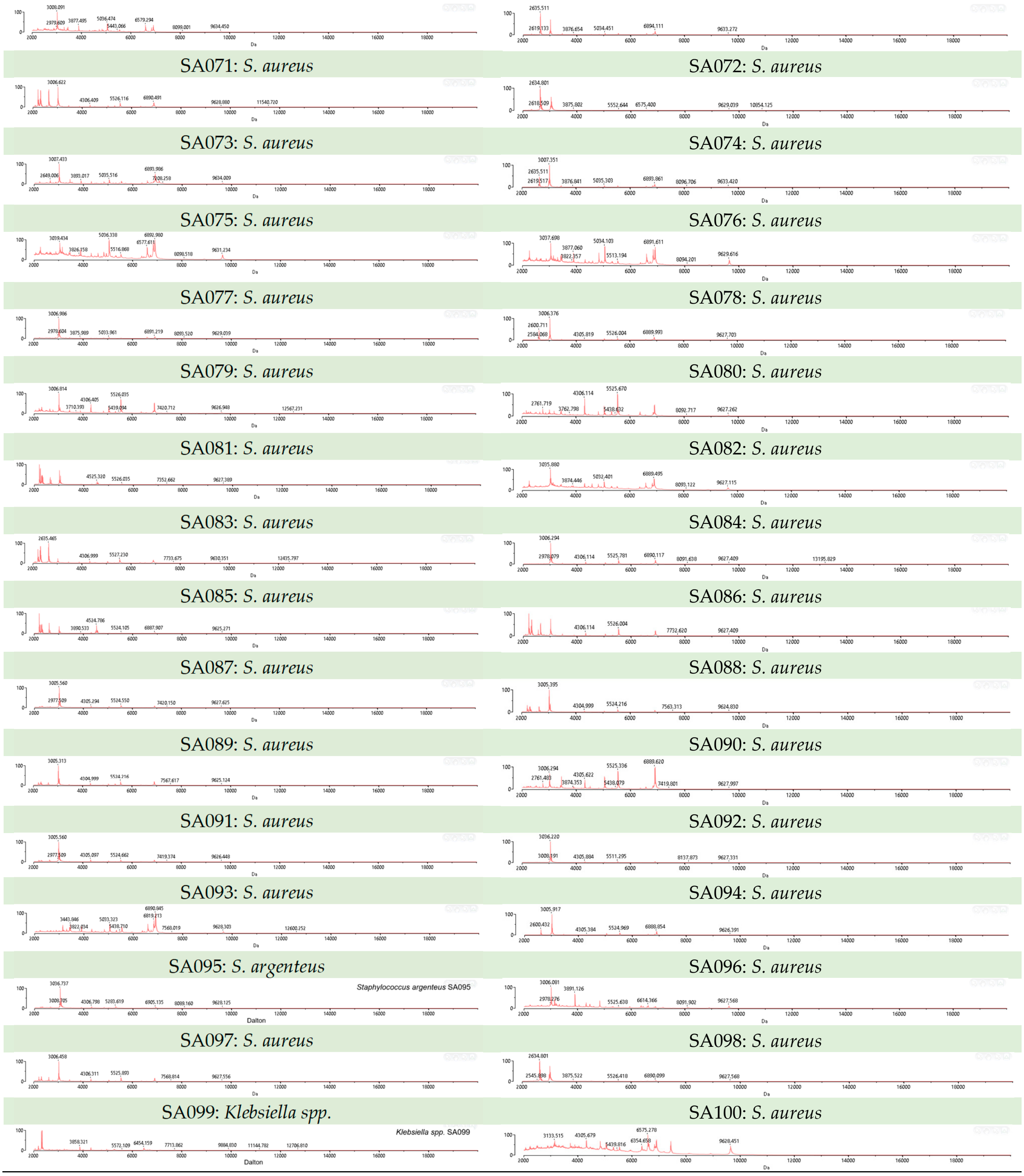
SA071: S. aureus
SA072: S. aureus
SA073: S. aureus
SA074: S. aureus
SA075: S. aureus
SA076: S. aureus
SA077: S. aureus
SA078: S. aureus
SA079: S. aureus
SA080: S. aureus
SA081: S. aureus
SA082: S. aureus
SA083: S. aureus
SA084: S. aureus
SA085: S. aureus
SA086: S. aureus
SA087: S. aureus
SA088: S. aureus
SA089: S. aureus
SA090: S. aureus
SA091: S. aureus
SA092: S. aureus
SA093: S. aureus
SA094: S. aureus
SA095: S. argenteus
SA096: S. aureus
Staphylococcus argenteus SA095
SA097: S. aureus
SA098: S. aureus
SA099: Klebsiella spp.
SA100: S. aureus
Klebsiella spp. SA099
